# Notfallausweis, Notfallmedikation und Informationsmaterial zur Prävention und Therapie der Nebennierenkrise (Addison-Krise): Ein österreichisches Konsensusdokument

**DOI:** 10.1007/s41969-022-00155-2

**Published:** 2022-03-02

**Authors:** Stefan Pilz, Michael Krebs, Walter Bonfig, Wolfgang Högler, Anna Hochgerner, Greisa Vila, Christian Trummer, Verena Theiler-Schwetz, Barbara Obermayer-Pietsch, Peter Wolf, Thomas Scherer, Florian Kiefer, Elke Fröhlich-Reiterer, Elena Gottardi-Butturini, Klaus Kapelari, Stefan Schatzl, Susanne Kaser, Günter Höfle, Dietmar Schiller, Vinzenz Stepan, Anton Luger, Stefan Riedl

**Affiliations:** 1 Klinische Abteilung für Endokrinologie und Diabetologie, Universitätsklinik für Innere Medizin, Medizinische Universität Graz, Auenbruggerplatz 15, 8036 Graz, Österreich; 2 Abteilung für Endokrinologie und Stoffwechsel, Klinik für Innere Medizin III, Medizinische Universität Wien, Wien, Österreich; 3 Abteilung für Kinder- und Jugendheilkunde, Klinikum Wels-Grieskirchen, Wels, Österreich; 4 Universitätsklinik für Kinder- und Jugendheilkunde, Johannes Kepler Universität Linz, Linz, Österreich; 5 Selbsthilfegruppe Netzwerk AGS-Österreich und Selbsthilfebeauftragte des Ordensklinikum Linz, Linz, Österreich; 6 Klinische Abteilung für allgemeine Pädiatrie, Medizinische Universität Graz, Graz, Österreich; 7 Universitätsklinikum für Kinder- und Jugendheilkunde, Uniklinikum Salzburg, Salzburg, Österreich; 8 Abteilung für Kinder- und Jugendheilkunde, Medizinische Universität Innsbruck, Innsbruck, Österreich; 9 Univ. Klinik für Innere Medizin 1 , Medizinische Universität Innsbruck, Innsbruck, Österreich; 10 Abteilung für Innere Medizin, LKH Hohenems, Hohenems, Österreich; 11 4. Interne Abteilung, Ordensklinikum Barmherzige Schwestern, Linz, Österreich; 12 Abteilung für Innere Medizin, Krankenhaus der Elisabethinen, Graz, Österreich; 13 St. Anna Kinderspital, Universitätsklinik für Kinder- und Jugendheilkunde, Medizinische Universität Wien, Wien, Österreich

**Keywords:** Leitlinie, Akute Nebenniereninsuffizienz, Adrenogenitales Syndrom, AGS, Schulung, Cortisol, Guideline, Acute adrenal insufficiency, Congenital adrenal hyperplasia, CAH, Education, Cortisol

## Abstract

Ein wichtiges Ziel bei der Behandlung der Nebenniereninsuffizienz ist die Prävention der Nebennierenkrise (auch akute Nebenniereninsuffizienz oder Addison-Krise genannt). Um in Österreich eine bessere Implementierung sowie Harmonisierung der Maßnahmen zur Prävention und Therapie der Nebennierenkrise zu erreichen, wurde dieses Konsensusdokument erarbeitet. Folgende Maßnahmen werden grundsätzlich für alle Patient*innen mit Nebenniereninsuffizienz empfohlen und in diesem Manuskript ausführlich erörtert: 1. Versorgung mit einer Notfallkarte („steroid emergency card“) sowie evtl. auch mit einem Armband oder einer Halskette (oder Ähnlichem) mit medizinischem Alarmhinweis „Nebenniereninsuffizienz, benötigt Glukokortikoide“. 2. Versorgung mit einem Hydrocortison-Notfallkit zur Injektion (alternativ auch Suppositorien/Zäpfchen zur Notfallapplikation) sowie ausreichenden oralen Glukokortikoiddosen für Stresssituationen/Erkrankungen. 3. Schulung von Patient*innen und Angehörigen zur Steigerung der Glukokortikoidtherapie in Stresssituationen bzw. bei Erkrankungen („sick day rules“) und zur Selbstinjektion von Hydrocortison. 4. Versorgung mit einer Behandlungsleitlinie (Informationszettel) zur Prävention und Therapie der Nebennierenkrise, welche bei Bedarf auch dem Gesundheitspersonal gezeigt werden soll. 5. Versorgung mit einer Notfall-Telefonnummer des behandelnden endokrinologischen Teams und/oder medizinisch geschulter Betreuungspersonen bzw. Angehöriger. 6. Regelmäßige (vorzugsweise jährliche) Wiederholung der Schulungsmaßnahmen. Dieses Konsensusdokument beinhaltet auch ausführliche Empfehlungen für die perioperative Glukokortikoidtherapie sowie für diverse andere Stresssituationen.

## Abstract (en)

A central goal of the adrenal insufficiency management is the prevention of acute adrenal insufficiency (also known as adrenal crisis or Addison crisis). This consensus document was generated in order to achieve better implementation and harmonization of measures for the prevention and treatment of acute adrenal insufficiency in Austria. The following measures are generally recommended for all patients with adrenal insufficiency and are outlined in this manuscript: (1) Provision of a “steroid emergency card” and possibly also a medical alert bracelet or necklace (or similar identification). (2) Provision of a hydrocortisone injection kit (or alternative glucocorticoid preparations) for emergency use plus sufficient oral glucocorticoid doses for stress situations/illness. (3) Education of patients and relatives on glucocorticoid stress dosing and “sick day rules” as well as on self-injection of hydrocortisone. (4) Provision of a treatment guideline (information leaflet) for the prevention and therapy of the adrenal crisis, which should also be shown to healthcare staff if necessary. (5) Provision of an emergency phone number (contact details) of the responsible endocrine specialist team or other trained staff. (6) Reinforcement of patient education on a regular basis (preferably yearly). This consensus document also includes recommendations for glucocorticoid dosing in the perioperative setting as well as in various other stress situations.

## Einleitung

Die Nebenniereninsuffizienz (NNI) ist eine Erkrankung, bei der die Nebennieren unzureichende Mengen an Glukokortikoiden (v. a. Cortisol) und/oder Mineralokortikoiden produzieren [1–3]. Es wird zwischen einer primären (Pathologie der Nebennieren; bei Kindern ist die häufigste Ursache das adrenogenitale Syndrom [AGS] und bei Erwachsenen die Autoimmunadrenalitis/Morbus Addison), sekundären (Pathologie der Hypophyse) und tertiären NNI (Pathologie des Hypothalamus) differenziert. Die initialen klinischen Symptome einer NNI sind oft unspezifisch, u. a. Abfall der Leistungsfähigkeit, Müdigkeit, ungewollter Gewichtsverlust, Hypotonie (niedriger Blutdruck), Übelkeit, abdominale Beschwerden und teils auch Hyperpigmentierung bei adrenaler Ursache. Die korrekte Diagnosestellung erfolgt oft erst nach einigen Jahren und wird häufig dadurch erschwert, dass sich die Symptome meist nur langsam entwickeln (Ausnahme u. a. Salzverlustsyndrom bei AGS und Schädel-Hirn-Trauma). Zentraler Bestandteil der Therapie der NNI ist die Hormonersatztherapie mit Glukokortikoiden (v. a. Cortisol), welche zum Ziel hat, die physiologischen Cortisolschwankungen mit tageszeitlicher Rhythmik, d. h. mit morgens hohen und abends niedrigeren Cortisolwerten, sowie Cortisolanstiegen in Stresssituationen z. B. bei Erkrankungen zu imitieren [1–3]. Neben der Glukokortikoidersatztherapie bzw. Glukokortikoidsubstitution (im Weiteren einfach Glukokortikoidtherapie genannt) müssen bei primärer NNI meistens auch Mineralokortikoide (v. a. Fludrocortison mit Handelsnamen Astonin H® oder Florinef®) ersetzt werden und bei Frauen fallweise auch Androgene in Form von DHEA (Dehydroepiandrosteron) [1–3].

Ein wichtiges Ziel bei der Behandlung der NNI ist die Prävention und Therapie der Nebennierenkrise (auch akute NNI oder Addison-Krise genannt) [4–9]. Patient*innen mit einer primären NNI haben ein höheres Risiko für eine Nebennierenkrise als Patient*innen mit sekundärer oder tertiärer NNI, und das Risiko ist besonders dann erhöht, wenn man erst vor kurzer Zeit eine Nebennierenkrise hatte. Die Nebennierenkrise ist ein lebensbedrohliches Zustandsbild, welches dadurch entsteht, dass dem Körper nicht genügend Cortisol (durch Eigenproduktion in den Nebennieren und/oder durch Substitution) zugeführt wird, um den aktuellen Cortisolbedarf ausreichend abzudecken. Die Amerikanische Gesellschaft für Endokrinologie („Endocrine Society“) definiert die Nebennierenkrise als einen medizinischen Notfall mit erniedrigtem Blutdruck (Hypotension), ausgeprägten abdominellen Beschwerden und deutlichen Laborveränderungen, der eine unmittelbare Therapie erfordert [3]. Die Definition der Nebennierenkrise ist somit unscharf und international bisher auch nicht einheitlich [3–9]. Eine häufig verwendete pragmatische Definition der Nebennierenkrise ist eine akute Beeinträchtigung des Gesundheitszustands in Verbindung mit absoluter Hypotension (systolischer Blutdruck < 100 mmHg) oder relativer Hypotonie (systolischer Blutdruck ≥ 20 mmHg niedriger als üblich), mit Veränderungen, die sich innerhalb von 1 bis 2 h nach Verabreichung von Glukokortikoiden deutlich verbessern (z. B. deutliche Besserung einer Hypotension innerhalb 1 h und Verbesserung der klinischen Symptome über einen Zeitraum von 2 h) [4]. Da bei Kindern die Diagnose einer Hypotonie im Notfall schwierig sein kann, ist eine pragmatische Definition der Nebennierenkrise bei Kindern eine akute Beeinträchtigung des Gesundheitszustandes in Verbindung mit akuten hämodynamischen Störungen (Hypotonie oder Sinustachykardie in Bezug auf die altersentsprechenden Normwerte) oder ausgeprägten Elektrolytwertveränderungen (z. B. Hyponatriämie und/oder Hyperkaliämie) und/oder Hypoglykämie, welche nicht durch andere Erkrankungen erklärbar sind, mit deutlicher Besserung durch eine parenterale Glukokortikoidzufuhr [4].

Eine Nebennierenkrise im Erwachsenenalter tritt in etwa bei 5–10 (in einer Studie 24) Patient*innen mit NNI innerhalb von 100 Patient*innenjahren auf und hat vermutlich eine Mortalitätsrate (Sterblichkeitsrate) von etwa 0,5 pro 100 Patient*innenjahren [5, 10–14]. Für das Kindesalter wurden ähnliche Zahlen publiziert [15–18], wobei rezente Studien bei Kindern marginal niedrigere Inzidenzen mit etwa 3–4 Nebennierenkrisen pro 100 Patient*innenjahren berichtet haben [19–22]. Patient*innen mit einer NNI haben langfristig ein etwas erhöhtes Mortalitätsrisiko, wobei in einer rezenten Studie ungefähr jeder 10. Todesfall durch eine Nebennierenkrise verursacht war [23–25]. Es kann jedoch postuliert werden, dass die meisten, wenn nicht sogar alle, Todesfälle durch eine Nebennierenkrise durch eine entsprechende Prävention und Therapie verhindert werden könnten [6].

Gemäß internationaler Richtlinien und Expertenrunden werden daher zur Prävention und Therapie der Nebennierenkrise folgende Maßnahmen für alle Patient*innen mit NNI empfohlen [3]:

1. Versorgung mit einer Notfallkarte („steroid emergency card“) sowie evtl. auch einem Armband oder einer Halskette (oder Ähnlichem) mit medizinischem Alarmhinweis „NNI, benötigt Glukokortikoide“.
2. Versorgung mit einem Hydrocortison-Notfallkit zur Injektion (alternativ auch andere Glukokortikoidpräparationen, z. B. Suppositorien/Zäpfchen zur Notfallapplikation) sowie mit ausreichenden oralen Glukokortikoiddosen für Stresssituationen/Erkrankungen.
3. Schulung von Patient*innen und Angehörigen zur Steigerung der Glukokortikoidtherapie in Stresssituationen bzw. bei Erkrankungen („sick day rules“) und zur Selbstinjektion des Hydrocortison-Notfallkits.
4. Informationszettel (Behandlungsleitlinie) zur Prävention und Therapie der Nebennierenkrise, welcher dem Gesundheitspersonal gezeigt werden soll.
5. Notfall-Telefonnummer des behandelnden endokrinologischen Teams und/oder medizinisch geschulter Betreuungspersonen bzw. Angehöriger.
6. Regelmäßige (vorzugsweise jährliche) Wiederholung der Schulungsmaßnahmen.

Ergänzend ist natürlich eine ausreichende Schulung und Ausbildung des Gesundheitspersonals in der Diagnostik, Prävention und Therapie der Nebennierenkrise wichtig. Trotz der in den letzten Jahren enormen Fortschritte in der Verbreitung der oben aufgelisteten Maßnahmen zur Prävention und Therapie der Nebennierenkrise bestehen weiterhin deutliche Defizite bzgl. der Schulung und Versorgung der Patient*innen sowie des aktiven Handelns von Patient*innen und Gesundheitspersonal im Falle einer drohenden oder bereits vorliegenden Nebennierenkrise [26–36]. In Österreich gibt es auf lokaler Ebene viele Initiativen, welche die Schulung und Versorgung der Patient*innen mit NNI in den letzten Jahren verbessert haben. Es findet sich aber eine große Heterogenität dieser Maßnahmen mit verschiedenen Notfallausweisen, Notfallinformationszetteln und Schulungsinitiativen. Mit der Anfrage, in diesem Kontext eine bessere Implementierung sowie Harmonisierung der Maßnahmen zur Prävention und Therapie der Nebennierenkrise in Österreich umzusetzen, ist die Selbsthilfegruppe Netzwerk AGS-Österreich an die Österreichische Gesellschaft für Endokrinologie (ÖGES) und an die Arbeitsgruppe der Pädiatrischen Endokrinologie und Diabetologie in Österreich (APEDÖ) der Österreichischen Gesellschaft für Kinder und Jugendheilkunde (ÖGKJ) herangetreten. Basierend auf einer systematischen Literaturrecherche in Pubmed (bis 28.05.2021) mit dem Suchbegriff „adrenal crisis“ wurde daher dieses Konsensusdokument erstellt, mit dem Ziel, Empfehlungen für die Prävention und Therapie der Nebennierenkrise für alle Altersgruppen zu erstellen und das dbzgl. Vorgehen in Österreich zu harmonisieren. Nach Erstellung einer ersten Dokumentversion durch Mitglieder des Vorstandes der ÖGES und der APEDÖ wurde diese zur Begutachtung auch diversen Mitgliedern der ÖGES und APEDÖ, der Selbsthilfegruppe Netzwerk AGS-Österreich sowie interessierten Patient*innen mit NNI zugesandt, wobei das finale Dokument dann unter Berücksichtigung der Gutachterkommentare durch den Vorstand der ÖGES und APEDÖ finalisiert wurde. Die dbzgl. Empfehlungen werden untenstehend aufgelistet und bedarfsweise deren Grundlage erörtert. Bezüglich der allgemeinen Diagnose- und Therapieempfehlungen, v. a. was die „chronische“ Dauertherapiesteuerung bei der NNI anbelangt, verweisen wir explizit auf die dbzgl. Richtlinien [1–3]. Da Hydrocortison das mit Abstand am häufigsten verordnete Glukokortikoid in der Therapie der NNI ist, beziehen wir uns in diversen Beispielen und Empfehlungen auf Hydrocortison, sind uns aber bewusst, dass manche Patient*innen auch mit anderen Glukokortikoiden therapiert werden. Bezüglich der Äquivalenzdosen der Glukokortikoide verweisen wir ebenfalls auf die entsprechende Fachliteratur, möchten aber dennoch anmerken, dass 20 mg Hydrocortison in etwa 5 mg Prednisolon bzw. 0,75 bis 0,8 mg Dexamethason entsprechen. Die synthetischen Glukokortikoide Prednisolon und Dexamethason haben aber eine im Vergleich zu Hydrocortison viel geringere mineralokortikoide („Aldosteron“) Wirkung.

Unsere in diesem Artikel publizierten Empfehlungen beziehen sich auf alle Formen der NNI, wobei wir uns primär auf Patient*innen mit ausgeprägter (schwerer) NNI fokussieren, weswegen bei milderen Formen der NNI natürlich auch ein differenzierteres, weniger progressives Vorgehen denkbar bzw. individuell umsetzbar ist. Wir möchten daher auch anmerken, dass wir zwar klare Handlungsempfehlungen publizieren, es aber natürlich je nach individueller Situation auch notwendig oder sinnvoll sein kann, davon etwas abzuweichen, v. a. da es in Österreich sehr unterschiedliche Zugangswege, Erreichbarkeiten und Kompetenzen der medizinischen Versorgung bzgl. NNI gibt, welche ein differenziertes Vorgehen erfordern. Obwohl wir mit diesem Dokument eine umfangreiche Richtlinie erstellt haben, können wir jedoch nicht vollständig auf alle speziellen Fragestellungen bzw. Aspekte in Stress- bzw. Notfallsituationen bei Patient*innen mit NNI detailliert eingehen.

## Versorgung mit einer Notfallkarte („steroid emergency card“) sowie einem Armband oder einer Halskette (oder Ähnlichem) mit medizinischem Alarmhinweis „NNI, benötigt Glukokortikoide“

Wir empfehlen, dass jeder Patient bzw. jede Patientin mit NNI immer (!) eine Notfallkarte („steroid emergency card“) bei sich trägt. Primär empfehlen wir dbzgl. die europäische Notfallkarte, welche sowohl für Erwachsene als auch für Kinder im Kreditkartenformat in zweisprachiger Ausführung (d. h. eine deutsch- und eine englischsprachige Seite) vorhanden ist. Diese Notfallkarte ist bereits von der Europäischen Gesellschaft für Endokrinologie („European Society of Endocrinology“) und der Europäischen Gesellschaft für pädiatrische Endokrinologie („European Society of Pediatric Endocrinology“) anerkannt und empfohlen (siehe https://adrenals.eu/emergency-card/international-emergency-cards/ zur Kartenansicht mit freier Download-Möglichkeit) [37]. Wir möchten jedoch bei dieser Notfallkarte für Kinder anmerken, dass dort in diversen Versionen Hydrocortison 25 mg für Kinder unter 1 Jahr und 50 mg für Kinder von 1–6 Jahren empfohlen ist, wir aber empfehlen gemäß internationaler Publikationen, dass 25 mg bei Kindern < 2 Jahre und 50 mg bei Kindern von 2–6 Jahren verabreicht werden [8]. Wir sehen es aber als durchaus vertretbar an, wenn bei Kindern zwischen 1 und 2 Jahren bereits die höhere Dosierung von 50 mg Hydrocortison im Rahmen einer vorliegenden oder drohenden Nebennierenkrise verabreicht wird, genauso wie wenn bei Kindern im Alter von 2 bis 3 Jahren nur 25 mg verabreicht werden (Anmerkung: Diesbezüglich finden sich auch in der Literatur teils inkonsistente Empfehlungen; [8, 15]). Die Versorgung der Patient*innen mit diesen Notfallkarten sollte, wenn möglich, über die behandelnden Ärzt*innen erfolgen. (Anmerkung: es gibt auch digitale Versionen solcher Ausweise, die zusätzlich verwendet werden könnten.)

Bezüglich der Versorgung mit einem Armband oder einer Halskette (oder Ähnlichem, wie z. B. Klammer am Sicherheitsgurt) mit dem medizinischen Alarmhinweis „NNI, benötigt Glukokortikoide“, gibt es diverse Versionen im Umlauf, welche online bestellt werden können. Hierzu geben wir keine Präferenz ab, empfehlen jedoch, diese zu tragen, sofern von den Patient*innen toleriert, wobei im Hinblick auf mögliche Freizeitunfälle z. B. Armbänder bei bestimmten Sportarten (z. B. Radfahren) sinnvoll sind. Die Armbänder/Halsketten sollten den Hinweis auf das Vorhandensein der NNI und die Notwendigkeit zur Verabreichung von Glukokortikoiden (oder Steroiden) beinhalten, optimalerweise auch ergänzt durch die Kontaktdaten eines endokrinologischen Teams und/oder medizinisch geschulter Betreuungspersonen bzw. Angehöriger.

## Versorgung mit einem Hydrocortison-Notfallkit zur Injektion (alternativ auch mit anderen Glukokortikoiden zur Notfallapplikation) sowie mit ausreichenden oralen Glukokortikoiddosen für Stresssituationen/Erkrankungen

Wir empfehlen, dass jeder Patient bzw. jede Patientin mit NNI immer einen Hydrocortison-Notfallkit (100 mg Hydrocortison) und/oder andere Glukokortikoidpräparationen, z. B. Suppositorien/Zäpfchen, zur Notfallapplikation in seinem bzw. ihrem unmittelbaren Umfeld oder zumindest verfügbar in der rasch erreichbaren Umgebung haben sollte. Der Notfallkit muss jedem Patienten bzw. jeder Patientin mit NNI von ärztlicher Seite verordnet oder direkt mitgegeben werden. Dieser Notfallkit beinhaltet 1 Ampulle Hydrocortison® 100 mg (anderer Handelsname z. B. Solu-Cortef® 100 mg), eine Einmalspritze (z. B. 2 ml), eine „dicke“ Kanüle/Nadel (z. B. rot G18) zur Entnahme von Hydrocortison aus der Ampulle, eine „dünne“ Kanüle/Nadel (z. B. orange G25 für subkutane [s.c.] Injektion und/oder z. B. G22 für intramuskuläre [i.m.] Injektion). Dieser beschriebene Notfallkit ist als illustratives Beispiel zu betrachten, wobei es natürlich je nach Gegebenheiten zu geringfügigen Abweichungen bei der Zusammenstellung kommen kann (z. B. anderer Handelsname für das Hydrocortisonpräparat, gering abweichende Nadelstärke/andere Nadelfarbe, evtl. bei Kindern zusätzlich auch ein Glukosegel, etc.).

Zusätzlich zum Hydrocortison-Notfallkit empfehlen wir, dass jeder Patient bzw. jede Patientin mit NNI immer auch ein paar Dosen (z. B. 3 Tagesdosen) der oral eingenommenen Glukokortikoidtherapie bei sich trägt bzw. rasch verfügbar hat, um diese in (unvorhergesehenen) Stresssituationen gleich einnehmen zu können [7].

## Schulung von Patient*innen und Angehörigen zur Steigerung der Glukokortikoidtherapie in Stresssituationen bzw. bei Erkrankungen („sick day rules“) und zur Hydrocortison-Selbstinjektion

Wir empfehlen, dass alle Patient*innen mit NNI, optimalerweise gemeinsam mit ihren nächsten Angehörigen vorzugsweise einmal pro Jahr bzgl. der notwendigen Steigerung der Glukokortikoidtherapie in Stresssituationen bzw. bei Erkrankungen („sick day rules“), zur Hydrocortison-Selbstinjektion mittels Notfallkits und zur Notwendigkeit der Inanspruchnahme ärztlicher Hilfe in Notfallsituationen durch das behandelnde Gesundheitspersonal (in der Regel die behandelnden Ärzt*innen) geschult werden bzw. das gelernte Wissen überprüft wird [3].

Bezüglich der sogenannten „sick day rules“ handelt es sich um Empfehlungen, die Glukokortikoiddosierung bei allen Altersgruppen in Stresssituationen bzw. bei Erkrankungen zu steigern, da in solchen Situationen bei Nebennieren-gesunden Menschen auch eine vermehrte körpereigene Cortisolsekretion erfolgt, welche für die diversen Körperfunktionen wie u. a. Unterdrückung einer überschießenden Inflammation (Entzündungsreaktion), Regulation des Stoffwechsels (z. B. Glukoneogenese) oder Aufrechterhaltung einer ausreichenden Kreislauffunktion wichtig ist [2, 5]. Der Cortisolbedarf in Stresssituationen wie z. B. Infekten ist auch deswegen erhöht, da sich dabei eine Glukokortikoidresistenz (Cortisolresistenz) ausbilden kann mit möglicher Entwicklung eines Teufelskreislaufs, bei dem durch Zytokinwirkungen die Glukokortikoidresistenz immer größer wird und durch die zu geringe Glukokortikoidwirkung die Zytokinfreisetzung nicht mehr ausreichend supprimiert werden kann [5]. Die folgenden Dosierungsempfehlungen basieren auf empirischen Expertenmeinungen und beziehen sich auf Maßnahmen, die von den Patient*innen selbst oder ihren Angehörigen nach entsprechender Schulung durchgeführt werden sollen [1–8].

Regel 1 („sick day rule 1“): Verdoppelung der täglichen Glukokortikoiddosis (z. B. statt exemplarisch Hydrocortison 10 mg-5 mg-5 mg dann Hydrocortison 20 mg-10 mg-10 mg) bei Erkrankungen mit Fieber und akuten Erkrankungen, welche eine Bettruhe und/oder eine Antibiotikatherapie erfordern (Anmerkung: Keine Dosiserhöhung bei nur „leichten“ Infekten wie z. B. etwas Rhinitis/Schnupfen ohne Fieber). Verdreifachung der täglichen Glukokortikoiddosis bei Fieber > 39 °C bzw. bei Kindern schon bei Fieber > 38 °C [2, 3, 5]. Alternative Empfehlungen, diese erhöhten Dosierungen etwas aufzurunden und gleichmäßig über den Tag zu verteilen oder sogar in extremen Stresssituationen alle 6 h zu verabreichen bzw. gleich bei Symptomen noch vor dem regulären Dosierungszeitpunkt eine Zusatzdosis einzunehmen, sehen wir bei Erwachsenen als individuell akzeptabel an. Bei Kindern mit z. B. Fieber über 38 °C oder in ähnlichen Stresssituationen empfehlen wir explizit die 4‑mal tgl. gleichmäßig (strikt alle 6 h!) verteilte und auf das Dreifache erhöhte Glukokortikoiddosis [15]. Begleitend soll unbedingt auf eine ausreichende Flüssigkeits‑, Elektrolyt- und Kohlenhydratzufuhr geachtet werden. Besonders bei Kindern soll auf eine genügende Kohlenhydratzufuhr geachtet werden und es ist dbzgl. empfehlenswert, wenn Eltern von Kindern mit NNI Glukosegels o. Ä. (z. B. Fruchtsaft) griffbereit haben (Anmerkung: die 4‑mal tgl. alle 6 h empfohlene Dosierung in Stresssituationen bei Kindern soll spezifisch dem v. a. nächtlichen Hypoglykämierisiko entgegenwirken, obwohl dies natürlich bedeutet, dass die Kinder zur Medikamenteneinnahme aufgeweckt werden müssen). Die erhöhten Glukokortikoiddosen sollten solange eingenommen werden, bis man sich schon deutlich besser fühlt, d. h. meistens für 2–3 Tage (kann aber auch länger z. B. eine Woche dauern); danach ist wieder die übliche Glukokortikoiddosis einzunehmen (oder alternativ noch für 1–2 Tage eine etwas erhöhte Tagesdosis). Aufgrund dieser nicht planbaren Dosissteigerungen sollte man immer so viele Tabletten zu Hause haben, dass man die Therapie für mindestens eine Woche in doppelter oder dreifacher Dosis einnehmen kann [7].

Regel 2 („sick day rule 2“): Bei schweren Erkrankungen, Unfällen, längerem Erbrechen und/oder Durchfall, beeinträchtigtem Bewusstseinszustand soll sich der erwachsene Patient/die Patientin die „Notfallspritze“ mit Hydrocortison 100 mg (bei Kindern 25, 50, oder 100 mg Hydrocortison je nach Angaben auf dem Notfallzettel) wenn möglich selbst verabreichen und danach sofort (!) ärztliche Hilfe aufsuchen, d. h. ins Krankenhaus fahren und ein endokrinologisches Team kontaktieren. Die Verabreichung der Notfallspritze kann auch durch geschulte Angehörige erfolgen. In jedem Fall muss aber begleitend auch rasch für eine ausreichende Flüssigkeits- und Elektrolytzufuhr (z. B. i.v. Kochsalzlösung) sowie ggf. (v. a. bei Kindern) für eine ausreichende Glukosezufuhr gesorgt werden.

Bei Kindern sollen natürlich die Eltern die „Notfallspritze“ mit Hydrocortison verabreichen. Alternativ können auch rektal zu verabreichende Prednisonsuppositorien („Zäpfchen“) bei den oben geschilderten Erkrankungszuständen verwendet werden. Diverse Experten raten zwar von der Verabreichung dieser Suppositorien v. a. bei Diarrhö (vermutlich geringe Resorption) eher ab, jedoch ist unserer Ansicht nach eine versuchsweise Verabreichung dieser Suppositorien bei Diarrhö oder ähnlichen Stresssituationen immer noch besser als keine Notfall-Glukokortikoidapplikation.

Zusätzlich zu den oben beschriebenen allgemeinen Empfehlungen gibt es noch ein paar spezielle Situationen bzw. Tipps für den Alltag, die wir als sinnvoll erachten [1–8, 38–40]:

Bei Erbrechen ist bei Erwachsenen sofort die zuvor eingenommene Glukokortikoiddosis in dann doppelter Dosis einzunehmen und für eine ausreichende Flüssigkeits- und Elektrolytzufuhr zu sorgen. Sollte dann innerhalb von 30 min wiederholt Erbrechen auftreten, ist die Hydrocortison-„Notfallspritze“ sofort zu verabreichen und danach sofort (!) ärztliche Hilfe aufzusuchen, d. h. ins Krankenhaus zu fahren und ein endokrinologisches Team zu kontaktieren [38]. Bei Kindern sollte bei Erbrechen ebenfalls die doppelte Hydrocortisondosis sofort nach dem Erbrechen eingenommen werden, aber wenn dann nochmals innerhalb von 30 min Erbrechen auftritt, kann bei ansonsten gutem Allgemeinzustand des Kindes auch ein Vorgehen mit Verabreichung eines Prednison-(Glukokortikoid‑)Suppositoriums gewählt werden, ohne gleich ärztliche Hilfe oder das Krankenhaus aufzusuchen, da es bei (v. a. jüngeren) Kindern relativ häufig zu nur kurzfristigen (z. B. ein paar Stunden dauernden) Episoden mit Erbrechen kommen kann ohne wesentliche sonstige Krankheitssymptomatik. Bei Kindern soll auch angemerkt werden, dass ein gewöhnliches „Aufstoßen“ keine Dosiserhöhung oder sonstige Handlung erforderlich macht.

Bei leichtem Durchfall ist die Glukokortikoid-Tagesdosis zu verdoppeln bzw. je nach Temperatur ggf. auch zu verdreifachen [38]. Dauert der Durchfall länger an und/oder wird schwerer oder es kommt Erbrechen hinzu, ist die Hydrocortison-„Notfallspritze“ sofort zu verabreichen (alternativ evtl. auch ein Prednisonsuppositorium) und danach ist sofort (!) ärztliche Hilfe aufzusuchen, d. h. ins Krankenhaus zu fahren und ein endokrinologisches Team zu kontaktieren. Bei Durchfall und/oder Erbrechen sollte auch unbedingt auf eine ausreichende orale Flüssigkeits‑, Elektrolyt- und Glukosezufuhr geachtet werden.

Bei nicht routinemäßig ausgeübten starken körperlichen Belastungen wie z. B. Marathon, Tageswanderung (v. a. bei heißen Temperaturen) empfehlen wir 30 bis 60 min vor der geplanten Aktivität die zusätzliche orale Einnahme von 5–10 mg Hydrocortison (optional bei Kindern > 6 Jahren z. B. 2,5 bis 5 mg und bei Kindern ≤ 6 Jahren z. B. 2,5 mg) sowie begleitend natürlich auch eine ausreichende Zufuhr von Kohlenhydraten sowie Flüssigkeits- und Elektrolytzufuhr [5, 39, 40]. Die Extradosis Hydrocortison kann bei längeren körperlichen Aktivitäten auch nach 2 bis 4 h wiederholt eingenommen werden [1]. Bei Kindern möchten wir aber betonen, dass ein gewöhnliches Spielen bzw. Herumtoben (auch wenn es mehrere Stunden andauert) in der Regel keine Dosiserhöhung erfordert, sofern es sich nicht um eine extreme Belastungssituation handelt.

Bei ausgeprägten psychischen Belastungssituationen wie z. B. Trauerfall oder große Prüfungen (z. B. Maturaprüfung) empfehlen wir auch (z. B. 30–60 min vor einer großen Prüfung) die zusätzliche orale Einnahme von 5–10 mg Hydrocortison bei Erwachsenen bzw. optional 2,5 bis 5 mg bei Kindern > 6 Jahren und 2,5 mg bei Kindern ≤ 6 Jahren [5, 40]. Sollten als übliche Glukokortikoidtherapie Medikamente mit verzögerter Wirkstofffreisetzung verwendet werden (z. B. Plenadren®, Efmody® oder Chronocort®), empfehlen wir, für notwendige akute Dosisanpassungen herkömmliche Hydrocortisonpräparate (z. B. Hydrocortone®, Hydrocortison®, Alkindi®) bevorzugt zu verwenden, da sie auch schneller wirksam sind.

Bei Aufenthalten in großer Hitze (z. B. bei Reisen) und/oder sportlichen Aktivitäten in großer Hitze kann in Einzelfällen auch eine leichte Dosiserhöhung von Fludrocortison sinnvoll sein. Eine vermehrte Salz- und Flüssigkeitszufuhr ist in solchen Situationen praktisch immer sinnvoll [1].

Patient*innen im Nachtschichtbetrieb sollen ihre Dosierung an ihren Arbeitsalltag bzw. Schlaf‑/Wachrhythmus anpassen, d. h. die übliche Morgendosis einfach dann einnehmen, wenn sie aufstehen [1].

Wir möchten auch klar betonen, dass bei einer vorliegenden NNI die Glukokortikoidtherapie natürlich niemals abgesetzt und/oder zeitweise pausiert werden darf!

Die Applikation der Hydrocortison-Notfallspritze kann i.m. oder auch s.c. erfolgen, da in einer Vergleichsstudie zwischen i.m.- und s.c.-Verabreichung nur eine geringe zeitliche Verzögerung des Cortisolanstiegs im Blut durch die s.c.-Injektion berichtet wurde [41]. Hydrocortison ist zwar für die s.c.-Applikation offiziell nicht zugelassen, es hat sich diese Applikationsart jedoch in der Praxis bewährt mit vergleichbarem klinischem Ansprechen wie bei der i.m.-Injektion und wahrscheinlich besserer Akzeptanz durch die Patient*innen [4, 28]. Obwohl international primär die i.m.-Injektion empfohlen wird, empfehlen und schulen wir auch die s.c.-Injektion für alle Patient*innen, die Hemmungen oder Vorbehalte gegenüber der i.m.-Injektion haben, sodass der Patient/die Patientin die Applikationsart wählen sollte, die als geeigneter empfunden wird. Es gibt deutschsprachige Videoclips sowohl für die i.m.- [42] als auch für die s.c.-Hydrocortisoninjektion [43]. Wichtig ist es uns zu betonen, dass die Verabreichung durch die Patient*innen (oder deren Angehörige) selbst erfolgen sollte und nicht erst nach zeitlicher Verzögerung durch das Gesundheitspersonal in z. B. einer Notaufnahme, da auch durch die Selbstinjektion vermutlich ein besseres Ergebnis (u. a. weniger stationäre Behandlungstage) für die Patient*innen erzielt werden kann [28].

Für alle in diesem Kapitel geschilderten Empfehlungen zur Dosissteigerung oder Applikation der Hydrocortisonspritze gilt der Leitsatz, dass im Zweifelsfall die Dosis gesteigert oder die Notfallspitze verabreicht werden soll, da es, auch aufgrund der kurzen Halbwertszeit von Hydrocortison, praktisch keine relevanten Nebenwirkungen bzw. Kontraindikationen einer dbzgl. möglicherweise lebensrettenden Akuttherapie gibt [6]. Natürlich kann aber eine chronisch (auf Dauer) zu hoch oder zu niedrig angesetzte Glukokortikoidtherapie zu Nebenwirkungen führen, wobei wir dbzgl. auf die entspr. Fachliteratur verweisen [1–3].

## Informationszettel (Behandlungsleitlinie) zur Therapie der Nebennierenkrise sowie Dosisanpassungen/Verhalten bei Stresssituationen/Operationen/medizinischen Eingriffen, welcher dem Gesundheitspersonal gezeigt werden soll

Wir empfehlen, dass jeder Patient bzw. jede Patientin mit NNI immer (!) einen Informationszettel (Behandlungsleitlinie) bei sich trägt mit Instruktionen für medizinisches Fachpersonal, wie eine Nebennierenkrise zu behandeln ist bzw. wie man bei medizinischen Eingriffen/Stresssituationen/Erkrankungen die Glukokortikoidtherapie dosieren sollte. Für diese Informationszettel für Erwachsene, Kinder > 6 Jahren, Kinder von 2 bis 6 Jahren und Kinder von 0 bis < 2 Jahren siehe die Abb. 1, 2, 3 und 4. Diese Informationszettel (Behandlungsleitlinien) sollen kein Ersatz für die Behandlung bzw. Beratung durch die betreuenden Ärzt*innen sein und sind beschränkt auf eine Auswahl von Verhaltensregeln in Stresssituationen bzw. bei Erkrankungen, die aus Platzmangel nicht detaillierter ausformuliert wurden, weswegen wir bei Unklarheiten in erster Linie auf den Volltext dieses Konsensusdokuments bzw. die jeweils betreuenden Ärzt*innen verweisen.

### Empfehlungen für Erwachsene

Eine Nebennierenkrise sollte schon bei Verdacht und ohne Verzögerung durch diagnostische Maßnahmen (z. B. Abwarten von Laborwerten) entsprechend behandelt werden. Empfohlen wird dabei ein sofortiger initialer Bolus mit Hydrocortison 100 mg i.v. (alternativ i.m., falls i.v. nicht möglich), gefolgt von Hydrocortison über einen Perfusor mit kontinuierlicher Infusionsrate von 200 mg pro 24 h (Perfusoren können mit z. B. Glukose 5 % oder NaCl 0,9 % vorbereitet werden) [1–8]. Statt einem Perfusor mit kontinuierlicher Infusionsrate kann alternativ auch Hydrocortison 50 mg i.v. (oder i.m. falls i.v. nicht möglich) alle 6 h verabreicht werden, wobei die kontinuierliche Infusion aufgrund pharmakokinetischer Studiendaten klar zu bevorzugen ist (Anmerkung: Cortisol hat eine Halbwertszeit im Blut von nur ca. 1,5 h) [44]. Bei klinischer Besserung (gewöhnlich nach ca. 24 h) kann dann statt der parenteralen Hydrocortisonverabreichung wieder die orale Hydrocortisontherapie in 2- bis 3‑facher Tagesdosis begonnen werden und diese dann schrittweise über 2–3 Tage auf die übliche Tagesdosis reduziert werden [4]. Fludrocortison (Astonin H® oder Florinef®), welches in Stresssituationen ohnehin nicht gesteigert werden muss, ist nicht erforderlich, solange die Hydrocortisontagesdosis mehr als 50 mg beträgt, weil Hydrocortison auch mineralokortikoide Wirkungen hat (40 mg Hydrocortison bewirken mineralokortikoide Effekte wie etwa 0,1 mg Fludrocortison) [2]. Somit muss Fludrocortison bei primärer NNI erst verabreicht werden, wenn die Hydrocortison-Tagesdosis unter 50 mg liegt, wobei schon typischerweise Fludrocortison hinzugegeben wird, wenn auf die orale Hydrocortisontherapie umgestellt wird [4]. Zusätzlich zur Hormonsubstitution muss auch eine Flüssigkeitssubstitution erfolgen mit initial 1000 ml NaCl 0,9 % i.v. innerhalb der ersten Stunde und dann weiterer Flüssigkeitssubstitution je nach Klinik (typischerweise jedoch insgesamt 3–4 l innerhalb der ersten 24 h; cave: Therapiesteuerung durch hämodynamisches Monitoring, um eine Übersubstitution zu vermeiden, mit begleitend auch regelmäßigen Serumelektrolytkontrollen, um z. B. einen zu raschen/starken Serumnatriumanstieg zu detektieren und entsprechend zu therapieren) [5, 8]. Es soll zudem die Blutglukose kontrolliert werden mit bei Hypoglykämie auch zusätzlicher i.v.-Infusion von z. B. 5 % Glukoselösung [4]. Neben einer intensivmedizinischen Überwachung sollten natürlich auslösende Ursachen für die Nebennierenkrise diagnostiziert und therapiert werden (z. B. Antibiotikatherapie bei Infektionen) sowie niedrig dosiertes Heparin als Thrombembolieprophylaxe und eine Protonenpumpeninhibitortherapie als Stressulkusprophylaxe erwogen werden [4].

Bei erwachsenen Patient*innen soll, ähnlich wie bei der Therapie der Addison-Krise, bei großen Operationen mit Allgemeinnarkose, Traumen, IVF (In-vitro-Fertilisation) mit Entnahme der Eizellen, bei der Geburt (vaginale Entbindung oder Sectio Caesarea) oder bei Erkrankungen, welche einen Aufenthalt auf der Intensivstation erforderlich machen, Hydrocortison 100 mg i.v. (alternativ i.m., falls i.v. nicht möglich) als Bolus verabreicht werden (bei Operationen direkt vor der Narkoseeinleitung). Unmittelbar danach soll Hydrocortison über einen Perfusor mit kontinuierlicher Infusionsrate von 200 mg pro 24 h verabreicht werden. Statt einem Perfusor mit kontinuierlicher Infusionsrate kann alternativ auch Hydrocortison 50 mg i.v. oder i.m. alle 6 h verabreicht werden, wobei die kontinuierliche Infusion aufgrund pharmakokinetischer Studiendaten klar zu bevorzugen ist [44]. Die kontinuierliche i.v.-Hydrocortisoninfusion sollte solange fortgeführt werden, bis wieder eine normale orale Nahrungszufuhr gut möglich ist, mit dann Wiedereinleitung der üblichen Hydrocortisontherapie in doppelter Dosis für gewöhnlich 2 Tage, d. h. bis zur vollständigen klinischen Besserung, mit dann wieder der üblichen Dosierung (diese doppelte Dosis kann bei schneller klinischer Erholung auch nur für 1 Tag bzw. bei langsamer klinischer Erholung auch für ca. 1 Woche eingenommen werden) [2, 8]. Ziel dieser relativ hohen Hydrocortisondosierungen ist es nicht, den exakt gleichen Cortisolanstieg zu imitieren, welcher bei Nebennieren-gesunden Personen in diesen Situationen auftritt, sondern den maximalen Cortisolanstieg zu erreichen, welcher bei solchen oben beschriebenen Situationen z. B. bei unvorhergesehenen Komplikationen auftreten kann [5].

Bei Koloskopien bzw. ähnlichen Verfahren bei Erwachsenen mit Notwendigkeit der Vorbereitung mit Laxanzien (Abführmitteln) empfehlen wir die Vorbereitungsphase unter klinischer Kontrolle (in erster Linie im Krankenhaus) mit Verabreichung von Hydrocortison 100 mg i.v. (alternativ s.c. oder i.m.) mit begleitender i.v.-Flüssigkeitsinfusionen (z. B. Kochsalzinfusionen) zu Beginn der Einnahme des Abführmittels und dann unmittelbar vor der Koloskopie die nochmalige Verabreichung von Hydrocortison 100 mg i.v. (alternativ s.c. oder i.m.) (dies grundsätzlich auch bei der Gastroskopie empfohlen; alternativ als empirische Expertenempfehlung die Einnahme der doppelten Gesamttagesdosis ca. 1,5 h vor der Gastroskopie für geeignete Patient*innen [cave: Aspirationsgefahr] oder nur 50 mg Hydrocortison i.v. bei Gastroskopiebeginn je nach ärztlicher Einschätzung) [8, 9]. Danach sollte für 24 h die doppelte Hydrocortisondosis eingenommen werden und dann wieder die übliche Dosierung. Bei bestimmten Patient*innen (gute Compliance, eher geringer Glukokortikoidbedarf bei vor allem sekundärer/tertiärer NNI) kann man aber auch ein ambulantes Vorgehen wählen. (Anmerkung: Bei Kindern empfehlen wird ein ähnliches Vorgehen, allerdings mit teils adaptierter Hydrocortisondosis: d. h. 25 mg bei 0‑ bis <2‑jährigen Kindern [< 15 kg], 50 mg bei 2‑ bis 6‑jährigen Kindern [15 bis 25 kg] und 100 mg bei > 6-jährigen Kindern [> 25 kg]).

Bei kleinen zahnärztlichen Eingriffen oder sehr kleinen ambulanten Eingriffen ohne Narkose sollte bei allen Altersgruppen die übliche Hydrocortison-Morgendosis nochmals 1 h vor dem Eingriff zusätzlich eingenommen werden mit dann doppelter Hydrocortisondosis für 24 h und danach wieder der üblichen Dosierung [5, 8, 40]. Handelt es sich um minimal belastende medizinische Behandlungen, muss keine Adaptierung der Hormonersatztherapie vor der Behandlung erfolgen.

### Empfehlungen für Kinder

Therapie der Nebennierenkrise bei Kindern [4, 8, 15, 45]: Die Therapieprinzipien entsprechen denen von Erwachsenen, jedoch werden adaptierte Dosierungen verwendet. Empfohlen wird ein sofortiger initialer Bolus mit Hydrocortison i.v. (oder i.m., falls i.v. nicht möglich) in einer Dosierung von 50 (bis 100) mg pro m^2^ Körperoberfläche. Als Orientierungshilfe zur Abschätzung des Hydrocortisonbolus werden 25 mg bei 0‑ bis <2‑jährigen Kindern (< 15 kg), 50 mg bei 2‑ bis 6‑jährigen Kindern (15 bis 25 kg) und 100 mg bei > 6-jährigen Kindern (> 25 kg) empfohlen [8]. Danach sollte Hydrocortison über einen Perfusor mit kontinuierlicher Infusionsrate von 50 bis 100 mg pro m^2^ Körperoberfläche pro 24 h verabreicht werden (d. h. ungefähr das 1‑ bis 2‑Fache des initial verabreichten Bolus über 24 h), alternativ kann auch Hydrocortison in einer Dosis von 12,5 bis 25 mg pro m^2^ Körperoberfläche i.v. oder i.m. alle 6 h verabreicht werden, wobei die kontinuierliche Infusion über Perfusor zu bevorzugen ist [4]. Bei klinischer Besserung (gewöhnlich nach ca. 24 h) kann statt der parenteralen Hydrocortisonverabreichung wieder die orale Hydrocortisontherapie in 2- bis 3‑facher Tagesdosis begonnen werden und diese dann schrittweise über 2–3 Tage auf die übliche Tagesdosis reduziert werden [4]. Zusätzlich zur Hormonsubstitution soll auch eine Flüssigkeitssubstitution erfolgen mit initial NaCl 0,9 % 20 ml pro kg Körpergewicht als Bolus i.v. und weiterer Flüssigkeitssubstitution je nach Klinik/Bedarf bzw. bei Schock bis 60 ml/kg innerhalb einer Stunde [3, 4, 8]. Regelmäßige (initial stündliche) Glukosekontrollen sind empfohlen plus bei Hypoglykämie auch 5 % oder 10 % Glukose i.v. (z. B. 2–5 ml/kg Körpergewicht einer 10%igen Glukose i.v.), wobei diverse Zentren auch schon präventiv (noch vor evtl. Auftreten einer Hypoglykämie) eine 5%ige Glukoseinfusion verabreichen [4, 8]. Begleitmaßnahmen wie intensivmedizinische Überwachung, Serumelektrolytkontrollen etc. sind wie beim Erwachsenen empfohlen.

Bei Kindern mit NNI empfehlen wir folgendes Vorgehen bei Operationen bzw. medizinischen Eingriffen [9]: Bei großen Operationen mit allgemeiner oder regionaler Anästhesie (Narkose): Bei der Narkoseeinleitung soll ein Hydrocortisonbolus mit 2 mg pro kg Körpergewicht (entsprechend in etwa 50 mg pro m^2^ Körperoberfläche) i.v. verabreicht werden, gefolgt von einer gewichtsbasierten kontinuierlichen Hydrocortisoninfusion über einen Perfusor während der Operation mit: 25 mg pro 24 h bei Gewicht bis 10 kg, 50 mg pro 24 h bei Gewicht von 11 bis 20 kg sowie bei > 20 kg, 100 mg pro 24 h bei präpubertären Kindern und 150 mg pro 24 h bei postpubertären Kindern [9]. Postoperativ sollen diese Hydrocortisoninfusionen weitergeführt werden oder es kann alternativ auch Hydrocortison 2 mg pro kg Körpergewicht (entsprechend in etwa 50 mg pro m^2^ Körperoberfläche) alle 4 h i.v. oder i.m. verabreicht werden bis zur klinischen Besserung bzw. bis wieder eine normale orale Nahrungszufuhr gut möglich ist [9]. Dann sollte die übliche orale Hydrocortisondosis in doppelter Dosierung für 48 h eingenommen werden mit dann schrittweiser Dosisreduktion auf die übliche Hydrocortisondosis innerhalb einer Woche [9].

Bei kleinen Eingriffen (Operationen), welche jedoch auch eine allgemeine Anästhesie (Narkose) erfordern, soll bei Kindern bei der Narkoseeinleitung ein Hydrocortisonbolus mit 2 mg pro kg Körpergewicht (entsprechend in etwa 50 mg pro m^2^ Körperoberfläche) i.v. oder i.m. verabreicht werden [9]. Danach sollte die orale Hydrocortisondosis für 24 h verdoppelt werden und dann wieder die übliche Dosierung eingenommen werden [9]. Sofort bei Aufnahme der oralen Zufuhr sollte auch bei primärer NNI die sonst übliche Fludrocortisondosis verabreicht werden [9].

Bei kleinen Eingriffen (Operationen) ohne Notwendigkeit zur allgemeinen Anästhesie (Narkose) soll zuvor (z. B. 30 min vor dem Eingriff) die doppelte Hydrocortison-Morgendosis oral eingenommen werden [9]. Nach dem Eingriff bei gutem Allgemeinzustand Fortführung der üblichen Therapiedosierungen [9].

Bei Kindern mit NNI sollte zudem ganz besonders auf regelmäßige perioperative Glukosebestimmungen geachtet werden, da Kinder besonders zu Problemen im Glukosestoffwechsel (cave: Hypoglykämie) neigen [9]. Perioperative Nüchternphasen sollten daher so gut wie möglich minimiert und Kinder mit NNI sollten daher auch auf Routine-Operationsplänen priorisiert werden [9].

## Notfall-Telefonnummer des behandelnden endokrinologischen Teams

Wir empfehlen, dass jeder Patient bzw. jede Patientin mit NNI immer (!) eine Notfall-Telefonnummer oder sonstige Kontaktdaten des behandelnden endokrinologischen Teams (oder zumindest von entsprechend geschulten Betreuungspersonen bzw. Angehörigen) bei sich hat und ggf. diese von den behandelnden Ärzt*innen mitgeteilt bekommt, z. B. vermerkt am Informationszettel zur Therapie der Nebennierenkrise (dbzgl. ist uns natürlich bewusst, dass lokale endokrinologische Teams oft nicht eine 24-Stunden-Erreichbarkeit gewährleisten können) [3].

## Regelmäßige (vorzugsweise jährliche) Wiederholung der Schulungsmaßnahmen

Wir empfehlen, dass jeder Patient bzw. jede Patientin bzw. auch die Eltern von Kindern mit NNI vorzugsweise jährlich von den behandelnden Ärzt*innen bzw. dem endokrinologischen Team bzgl. der Maßnahmen zur Prävention und Therapie der Nebennierenkrise geschult werden und das dbzgl. Wissen bei den Patient*innen bzw. Eltern auch kontrolliert wird [3]. Die Prognose bzw. die übrigen Komorbiditäten der Patient*innen sollten, ähnlich wie bei anderen diagnostischen und therapeutischen Maßnahmen, bei Überlegungen zur Schulungsintensität- und -modalität selbstverständlich auch einbezogen werden, sodass ein evtl. differenzierteres, individualisierteres Vorgehen sinnvoll sein kann.

## Klinische Daten zur Nebennierenkrise bzw. Stressdosisanpassung

Die häufigsten Ursachen einer Nebennierenkrise bei Erwachsenen sind Erbrechen und Durchfall (initiale Symptome), gefolgt von bakteriellen oder viralen Infektionskrankheiten (z. B. Atemwegsinfekte, Harnwegsinfekte, etc.) [4, 5, 45]. Manchmal können auch besonders schwerwiegende psychische/emotionale Stresssituationen Auslöser sein, genauso wie auch unregelmäßige oder gestoppte Hormonersatztherapie [4, 45]. Andere Ursachen mit erhöhter körperlicher Stressbelastung (z. B. Unfälle/Traumen, etc.) wurden auch als mögliche Auslöser einer Nebennierenkrise beschrieben, genauso wie Medikamente, welche den Cortisolabbau erhöhen (z. B. Phenobarbital, Phenytoin, Schilddrüsenhormone oder Carbamazepin, etc.), aber manchmal kann auch kein direkter Triggerfaktor identifiziert werden [45].

Die klinische Symptomatik einer Nebennierenkrise entwickelt sich meistens über mehrere Stunden, wobei durchschnittlich etwa 24 h zwischen den ersten Symptomen und der voll ausgebildeten Nebennierenkrise liegen [45]. Eine Auswahl häufiger und typischer Symptome, die auf eine beginnende Nebennierenkrise hindeuten können, sind z. B. allgemeine Schwäche, Müdigkeit, Übelkeit, Erbrechen, Bauchschmerzen, Tachykardie, vermehrtes Schwitzen, Fieber, Bewusstseinseintrübungen, Verwirrtheit, etc. [4, 45].

Typische, aber nicht immer vorhandene Laborbefunde einer Nebennierenkrise bei Erwachsenen sind z. B. eine Hyponatriämie, welche vor allem durch die fehlende Cortisolsuppression des antidiuretischen Hormons (ADH; Vasopressin) sowie durch einen Natriumverlust im Harn aufgrund des Mineralokortikoidmangels verursacht wird (dieser ist aber nur bei den meisten Formen der primären NNI relevant) [45]. Der Mineralokortikoidmangel kann aber auch zu einer (potenziell lebensbedrohlichen) Hyperkaliämie führen. Eine metabolische Azidose sowie eine eingeschränkte Nierenfunktion aufgrund der Hypovolämie/Hypotonie ist ebenfalls häufig vorhanden. Eine Hypoglykämie tritt häufiger bei Kindern auf.

Bei Kindern, bei denen Nebennierenkrisen am häufigsten vor dem 10. Lebensjahr auftreten, sind die häufigsten Ursachen gastrointestinale Infekte (Brechdurchfälle), Atemwegsinfekte sowie diverse andere Infektionen wie z. B. Harnwegsinfekte. Typische Symptome einer beginnenden Nebennierenkrise bei Kindern sind Übelkeit, Erbrechen, Durchfall, Dehydrierung, Fieber, Schüttelfrost, Müdigkeit und Bauchschmerzen [45]. Symptome einer Hypoglykämie wie z. B. Schwitzen, Verwirrtheit, Blässe, Tachykardie, Krämpfe etc. können aber auch vorhanden sein. Im Laborbefund können sich neben einer häufig vorhandenen Hypoglykämie auch eine Hyponatriämie, Hyperkaliämie und metabolische Azidose finden. Gerade bei Kindern müssen spezielle Aspekte auch in der Schulung sowie der Genese der NNI (meistens handelt es sich um ein AGS) beachtet werden, auf die wir jedoch aus Platzgründen nicht eingehen können [46–50]. Ein empfehlenswerter Vortrag über Nebennierenkrisen und Notfallmanagement bei AGS für Patient*innen bzw. Angehörige findet sich in Referenz [51].

Während einer Schwangerschaft kommt es zu einem etwas zunehmenden Cortisolbedarf bei u. a. ansteigendem Cortisolbindendem Globulin, weswegen eine 20- bis 40%ige Erhöhung der tgl. Hydrocortisondosis (d. h. meistens Erhöhung um ca. 5–10 mg) bei Patientinnen mit NNI im dritten Trimester empfohlen wird [3, 52–54]. Die Mineralokortikoiddosis kann während der Schwangerschaft meistens unverändert weitergenommen werden, wobei es vereinzelt auch notwendig sein kann, Fludrocortison etwas zu steigern, da Progesteron auch eine antimineralokortikoide Wirkung hat und somit z. B. zu vermehrter orthostatischer Hypotension führen kann [52, 54]. Eine rezente Übersichtsarbeit berichtete bei 7 % aller Schwangerschaften über eine Nebennierenkrise, wobei sich unter regelmäßiger endokrinologischer Kontrolle (d. h. klinische Kontrolle in jedem Trimester) in den allermeisten Fällen ein für Mutter und Kind unkomplizierter Schwangerschaftsverlauf zeigt [54]. Die Dosierungsempfehlungen für Hydrocortison während der Geburt oder dem Kaiserschnitt (Sectio Caesarea) wurden bereits weiter oben beschrieben.

Bei älteren Patient*innen mit NNI kommen als komplizierende Faktoren hinzu, dass häufig auch diverse Komorbiditäten vorliegen und es auch zunehmende Unzulänglichkeiten bzgl. der Schulung bzw. Implementierung von Präventionsstrategien in Bezug auf die Nebennierenkrise gibt [55].

Die Vorhersage von Nebennierenkrisen ist sehr schwierig, jedoch zeigen rezente Untersuchungen, dass insbesondere ein niedriges Serumnatrium sowie hohes C‑reaktives Protein (CRP) Hinweise auf ein höheres Risiko für eine Nebennierenkrise sind [56].

Bezüglich alternativer Glukokortikoidapplikationswege hat eine Studie bei erwachsenen Patientinnen mit Morbus Addison gezeigt, dass eine rektale oder vaginale Applikation von Prednisonsuppositorien einer subkutanen oder intramuskulären Hydrocortisongabe deutlich unterlegen war und somit solche Suppositorien nicht primär empfohlen werden können [57]. Bei Kindern (und auch Erwachsenen) können aber rektal angewandte Suppositorien dann erwogen werden, wenn ansonsten keine andere Applikation eines Glukokortikoids möglich ist. Entsprechend der schlechteren Bioverfügbarkeit kann in diesem Fall die relativ hohe Dosis von 100 mg Prednison als rektal angewandtes Suppositorium bei allen Altersgruppen verabreicht werden.

Bezüglich der zirkadianen Rhythmik des Cortisols mit v. a. morgens sehr hohen Konzentrationen kann diese Rhythmik bei extremen Stresssituationen, wie z. B. bei Patient*innen auf einer Intensivstation, aufgehoben sein, wobei sich bei moderateren Stresssituationen wie z. B. Infekten (ohne schwere Sepsis) oder Herzkatheteruntersuchungen weiterhin eine entsprechende, wenn auch möglicherweise etwas beeinträchtigte, Tagesrhythmik zeigt [58–61]. Dies ist insofern relevant, als dass wir bei moderaten Stresssituationen eine Dosiserhöhung unter Beibehaltung der Tagesrhythmik empfehlen, jedoch bei massivem Stress wie z. B. einer Addison-Krise und v. a. bei Säuglingen und Kleinkindern (dies auch im Hinblick auf das Hypoglykämierisiko) eine gleichmäßig über den Tag verteilte erhöhte Glukokortikoiddosis empfehlen.

Bezüglich der SARS-CoV-2- bzw. COVID-19-Impfung wird von internationalen Gesellschaften empfohlen, dass den Impfempfehlungen für die Allgemeinbevölkerung gleichermaßen Patient*innen mit NNI folgen sollten [62]. Die Glukokortikoiddauertherapie soll gemäß der Mehrheit der Experten und auch unserer Empfehlung nach bei der Impfung unverändert fortgeführt werden, und es soll natürlich bei Nebenwirkungen (z. B. Fieber) eine sonst übliche Stressdosiserhöhung erfolgen. (Anmerkung: ca. 1/3 aller Experten empfiehlt jedoch eine automatische passagere Dosiserhöhung der Glukokortikoidtherapie um den Impfzeitpunkt [63], ein Vorgehen, das wir zwar nicht empfehlen, aber als akzeptabel bei individuellen Patient*innen ansehen.) Das Triggern einer Addison-Krise durch eine Impfung ist zwar in der Literatur beschrieben, aber dies ist eine absolute Rarität [64, 65].

## Zusammenfassung und Schlussfolgerungen

Maßnahmen zur Prävention, raschen Erkennung und Therapie der Nebennierenkrise sind wichtig, um die Morbidität und Mortalität bei Patient*innen mit NNI zu reduzieren. Schulungsprogramme für Patient*innen mit NNI (optimalerweise gemeinsam mit deren Angehörigen), wie in Deutschland erfolgreich evaluiert, sind dabei eine wichtige Maßnahme, damit Patient*innen besser bei ihrer eigenen Therapie mitwirken können („Patient Empowerment“) [29, 66]. Ebenso ist es wichtig, die Ausbildung des Gesundheitspersonals in Bezug auf die Diagnostik und Therapie der Nebennierenkrise weiter zu optimieren, da auch hier Defizite vorliegen [67]. Eine gegenseitig wertschätzende und zielorientierte Kommunikation zwischen sehr gut geschulten und informierten Patient*innen (samt Angehörigen) und dem medizinischen Fachpersonal, welches nicht immer sehr gut bezüglich Diagnostik und Therapie der Nebennierenkrise ausgebildet ist, erscheint dabei auch von zentraler Bedeutung.

Zukünftige wünschenswerte und anzustrebende Entwicklungen wären ein Hydrocortison-Notfallpen, eine Erfassung der österreichischen Patient*innen mit NNI in einem nationalen und/oder internationalen Register sowie auch eine Verlinkung der Patient*innendaten mit den Rettungs- bzw. Notfallzentralen, sodass der Hinweis auf das Vorliegen einer NNI auch automatisch bei Einsätzen/Notfällen übermittelt wird. Ebenso wäre es zu empfehlen, dass in Rettungs- und Notfallsystemen flächendeckend das Hydrocortison-Notfallset vorhanden ist sowie ein strukturiertes Schulungsprogramm für Patient*innen mit NNI auch in Österreich (ähnlich wie schon in Deutschland) besser etabliert wird. Regelmäßige Re-Evaluierungen und ggf. zukünftige Adaptierungen dieses Konsensdokuments sind ebenfalls für die nächsten Jahre geplant.
